# Apelin prevents diabetes-induced poor collateral vessel formation and blood flow reperfusion in ischemic limb

**DOI:** 10.3389/fcvm.2023.1191891

**Published:** 2023-08-11

**Authors:** Stéphanie Robillard, Kien Trân, Marie-Sophie Lachance, Tristan Brazeau, Elizabeth Boisvert, Farah Lizotte, Mannix Auger-Messier, Pierre-Luc Boudreault, Éric Marsault, Pedro Geraldes

**Affiliations:** ^1^ Research Center of the Centre Hospitalier Universitaire de Sherbrooke, Sherbrooke, QC, Canada; ^2^ Department of Pharmacology and Physiology, Université de Sherbrooke, Sherbrooke, QC, Canada; ^3^ Division of Cardiology, Department of Medicine, Université de Sherbrooke, Sherbrooke, QC, Canada; ^4^ Division of Endocrinology, Department of Medicine, Université de Sherbrooke, Sherbrooke, QC, Canada

**Keywords:** peripheral arterial disease, angiogenesis, diabetes, apelinergic system, apelin

## Abstract

**Introduction:**

Peripheral arterial disease (PAD) is a major risk factor for lower-extremity amputation in diabetic patients. Unfortunately, previous clinical studies investigating therapeutic angiogenesis using the vascular endothelial growth factor (VEGF) have shown disappointing results in diabetic patients, which evokes the necessity for novel therapeutic agents. The apelinergic system (APJ receptor/apelin) is highly upregulated under hypoxic condition and acts as an activator of angiogenesis. Apelin treatment improves revascularization in nondiabetic models of ischemia, however, its role on angiogenesis in diabetic conditions remains poorly investigated. This study explored the impact of Pyr-apelin-13 in endothelial cell function and diabetic mouse model of hindlimb ischemia.

**Methods:**

Nondiabetic and diabetic mice underwent femoral artery ligation to induce limb ischemia. Diabetic mice were implanted subcutaneously with osmotic pumps delivering Pyr-apelin-13 for 28 days. Blood flow reperfusion was measured for 4 weeks post-surgery and exercise willingness was assessed with voluntary wheels. In vitro, bovine aortic endothelial cells (BAECs) were exposed to normal (NG) or high glucose (HG) levels and hypoxia. Cell migration, proliferation and tube formation assays were performed following either VEGF or Pyr-apelin-13 stimulation.

**Results and Discussion:**

Following limb ischemia, blood flow reperfusion, functional recovery of the limb and vascular density were improved in diabetic mice receiving Pyr-apelin-13 compared to untreated diabetic mice. In cultured BAECs, exposure to HG concentrations and hypoxia reduced VEGF proangiogenic actions, whereas apelin proangiogenic effects remained unaltered. Pyr-apelin-13 induced its proangiogenic actions through Akt/AMPK/eNOS and RhoA/ROCK signaling pathways under both NG or HG concentrations and hypoxia exposure. Our results identified the apelinergic system as a potential therapeutic target for angiogenic therapy in diabetic patients with PAD.

## 1. Introduction

Peripheral arterial disease (PAD) is defined as a complete or partial atherosclerotic occlusion in the artery, which reduces blood supply to the limbs. As the disease progress, critical limb ischemia (CLI) and ischemic ulceration of the foot can occur, which increase the risk of limb amputation. Diabetes and smoking are the strongest risk factors for the development of PAD (1). In individuals with diabetes, PAD appears at an earlier age, progresses more rapidly, and is more severe, which makes this population 5 times more susceptible to lower extremity amputation (2). Indeed, the combination of PAD and diabetes is responsible for 54% of nontraumatic amputations (3) and the 5-year survival rate following amputation is 40% (4). These poor clinical outcomes are partly due to altered angiogenic processes impairing collateral vessel formation, a compensatory mechanism in response to tissue hypoxia. Diabetes causes an imbalance in the production of reactive oxygen species (ROS) (5), nitric oxide (NO) (6) and growth factors such as the vascular endothelial growth factor (VEGF) (7) and the platelet-derived growth factor (PDGF) (8), contributing to endothelial cell dysfunction. VEGF has been the most studied growth factor for therapeutic angiogenesis and revealed promising results in preclinical studies. However, randomized phase II clinical trials using VEGF gene therapy failed to reduce amputation rate and had no benefits on quality-of-life measurements (9). These disappointing outcomes could be attributed to diabetes-induced growth factor resistance, inhibiting proangiogenic signaling in endothelial and smooth muscle cells (10). Therefore, it is crucial to pursue preclinical research for new targets that are not affected by the hyperglycemic milieu to ensure proper collateral vessel formation and limit the incidence of CLI and limb amputation.

Apelin and its receptor APJ, a G protein-coupled receptor (GPCR), are expressed in multiple tissues such as the heart, brain, kidney, adipose tissue and endothelium (11). Being widely distributed in the body, apelin/APJ modulate multiple physiological processes including cardiac inotropy, blood pressure, glucose metabolism, water homeostasis and angiogenesis (12). The main apelin fragments generated by the cleavage of preproapelin are apelin-13, apelin-17 and apelin-36, and they all differently regulate APJ signal transduction pathways, causing different biological effects (13, 14). Apelin-13 can be further post-translationally modified by a cyclization of the glutamine in N-terminus to produce pyroglutamylated apelin-13 (Pyr-apelin-13) (15). Compared to the other apelins, Pyr-apelin-13 is the most potent and abundant isoform in the plasma and cardiac tissue (16, 17) with enhanced plasma half-life (18). Apelin/APJ axis is essential for vascular homeostasis since it regulates the enlargement of new blood vessels during angiogenesis and regulates parallel alignment of arteries and veins in the skin (19). Furthermore, APJ has been shown to be required for normal development of the cardiovascular system since in APJ deficient mice, over fifty percent of embryos die during pregnancy and the survived embryos have insufficient vascular maturation and abnormal ventricular wall formation (20).

Apelin administration or apelin gene therapy has been reported to improve revascularization in nondiabetic model of hindlimb ischemia (21–23). However, to our knowledge, no study has investigated the impact of the apelinergic system (apelin/APJ axis) on angiogenesis under the combination of limb ischemia and diabetes. The present study further explored the contribution of Pyr-apelin-13 on angiogenesis in endothelial cells exposed to high glucose concentrations and hypoxia, and in diabetic mice following hindlimb ischemia.

## 2. Materials and methods

### 2.1. Reagents and antibodies

Primary antibodies for immunoblotting were purchased from commercial sources: GAPDH horseradish peroxidase (V-18), endothelial nitric oxide synthase (eNOS) (C-20) from Santa Cruz Biotechnology Inc (Dallas, TX, USA); protein kinase B (Akt) (9272S), phospho-Akt (193H12), AMP-activated protein kinase α (AMPKα) (2532S), p-AMPKα (2535S), p-eNOS Ser1177 (9571S), Ras homolog family member A (RhoA) (2117S), rho-associated coiled-coil-containing protein kinase (ROCK) 1 (4035S) and ROCK-2 (8236S), and secondary antibody of anti-rabbit (7,074 V) and anti-mouse (7076S) peroxidase-conjugated from Cell Signaling (Danver, MA, USA); anti-α smooth muscle actin (ab5694) and p-ROCK2 (ab228008) from Abcam (Toronto, ON, CA); anti-CD31 (558,736) and p-eNOS Ser633 (612,664) from BD Bioscience (Mississauga, ON, CA). Secondary antibodies for immunofluorescence FITC conjugated anti-rat IgG (712-095-153) and Alexa Fluor 647 conjugated anti-rabbit IgG (711-606-152) were purchased from Jackson ImmunoResearch Laboratories (West Grove, PA, USA). Halt Protease Inhibitor Cocktail (78,430) was purchased from Thermo Fisher Scientific (Waltham, MA, USA). SEP-COLUMN (RK-SEPCOL-1) for plasma peptides extraction and Apelin-12 (Human, Rat, Mouse, Bovine) Enzyme Immunoassay (EIA) (EK-057-23) were purchased from Phoenix Pharmaceuticals Inc (Burlingame, CA, USA). Fetal bovine serum (FBS, 080-150), penicillin-streptomycin (P/S, 450-201-EL) and phosphate-buffered saline (PBS, 311-410-CL) were purchased from Wisent Bioproducts (Saint-Jean Baptiste, QC, CA). Dulbecco's Modified Eagle Medium (DMEM) low glucose (31,600–034) were obtained from Invitrogen (Burlington, ON, CA). VEGF-A_165_ was purchased from R & D (293-VE-010, Minneapolis, MN, USA). Pyr-apelin-13 (pyr-Ape-13) was synthesized and generously provided by Dr. Boudreault's laboratory from the *Institut de Pharmacologie de Sherbrooke*. D-mannitol (M-120), sodium citrate (BP327.1) and citric acid (A940–500) were purchased from Fisher scientific (Hampton, NH, USA). All other reagents used, including streptozotocin (S0130), ethylenediaminetetraacetic acid (EDTA, E5134), bovine serum albumin (BSA, A7906) d-glucose (158,968), leupeptin (L2884), phenylmethylsulfonyl fluoride (P7626), aprotinin (A6279), NaF (S7920) and Na_3_VO_4_ (S6508) were purchased from Sigma-Aldrich (St. Louis, MO, USA).

### 2.2. Cell culture

Bovine aortic endothelial cells (BAECs) were isolated from fresh harvested aorta as previously described (7). Cells from passages 2 to 7 were trypsinized and cultured in DMEM 2.5% FBS and 1% penicillin-streptomycin. For all the *in vitro* experiments, cells were cultured in DMEM 0.1% FBS containing normal (NG; 5.6 mmol/L + 19.4 mmol/L of Mannitol) or high glucose (HG; 25 mmol/L) levels for up to 48 h and exposed to hypoxia for the last 16 h (1% O_2_) to reproduce the ischemic state of PAD. For immunoblotting and cell signaling experiments, BAECs were stimulated with either VEGF-A 10 ng/ml for 5 min (7) or Pyr-apelin-13 200 nM for 1 h, or 24 h prior to RNA extraction and quantitative PCR analysis. For migration, proliferation and tube formation assays BAECs were stimulated with either VEGF-A 25 ng/ml (7) or Pyr-apelin-13 100 nM. Concentrations used for Pyr-apelin-13 stimulation were based on previous dose dependent experiments (data not shown).

### 2.3. Migration assay

BAECs were trypsinized, counted and seeded at 20 000 cells inside the 2 wells of Ibidi's culture insert (80,209, Ibidi, Fitchburg, WI, USA) placed into a 12-well plate. After cell adhesion (4 h later), BAECs were exposed to NG or HG concentrations for 24 h. Following the 24 h treatment, each culture insert was removed, and the wells were filled with 1 ml of either NG or HG medium. Cells were then stimulated, or not, with either VEGF-A (25 ng/ml) or Pyr-apelin-13 (100 nM) and placed into the hypoxic incubator (1% O_2_) for 16 h. Cell migration was evaluated by taking images under Nikon eclipse Ti microscope at 10 × magnification immediately after removing the culture insert and at the end of the experiment (16 h later). Analysis was performed with Image J software by measuring the difference in occupied area immediately after insert removal and following 16 h stimulation in NG or HG condition. Results were reported as a % of cell migration for analysis.

### 2.4. Proliferation assay

BAECs were trypsinized, counted and seeded in a 96-well plate at 5 000 cells per well. After cell adhesion (4 h later), cells were exposed to NG or HG concentrations for 24 h, stimulated with either VEGF-A (25 ng/ml) or Pyr-apelin-13 (100 nM) and placed into the hypoxic incubator for 16 h. Cells were then fixed in 4% paraformaldehyde for 5 min, rinsed twice in PBS and incubated with the nuclear counterstain DAPI (D9542, Sigma-Aldrich, St. Louis, MO, USA) at 0.001 mg/ml for 10 min. Fluorescence microscopy and the NIS-Elements software of Nikon eclipse Ti microscope were used to visualize and count cells, reported as the number of cells/mm^2^ for analysis.

### 2.5. Lumen formation assay

BAECs were cultured in 100 mm petri dish and exposed to NG or HG concentrations for 24 h and then placed into the hypoxic incubator (1% O_2_) for 16 h. On the day of the assay, 10 μl of Matrigel Matrix (Growth Factor Reduced, 356,230, Corning, Glendale, AZ, USA) was applied into each well of Ibidi's μ-slide rack (81,506, Ibidi, Fitchburg, WI, USA) and incubated for 30 min at 37°C to allow polymerization. Cells were then trypsinized, counted, and 600 000 cells were seeded per well on top of the Matrigel. After seeding, cells were immediately stimulated, or not, with VEGF-A (25 ng/ml) or Pyr-apelin-13 (100 nM) and the μ-slide rack was placed into the hypoxic incubator for 4 h. At the end of the experiment (4 h later), the lumen formation was visualized, and images were taken under Nikon eclipse Ti microscope at 4 × magnification. Endothelial cell tube formation ability after NG or HG treatment and angiogenic factor stimulation was measured using Image J software by counting the number of formed lumens and normalized on the NG condition to present it as a fold increase.

### 2.6. Immunoblot analysis

Adductor muscles or endothelial cells were lysed in RIPA buffer containing protease inhibitors (1 mmol/L phenylmethylsulfonyl fluoride, 2 µg/ml aprotinin, 10 µg/ml leupeptin, 1 mmol/L Na3VO4, 1 mmol/L NaF) and protein concentrations were measured by DC kit (5,000,116, BioRad, Mississauga, ON, CA). The lysates (20 μg for BAECs and 50 μg for adductor muscles) were separated by SDS-PAGE and transferred to PVDF membranes, which were blocked with 5% skim milk for 1 h at room temperature. Membranes were incubated overnight with primary antibodies at a 1:1,000 dilution in 5% skim milk (or in 5% BSA for phospho-ROCK-2 and p-AMPKα), followed by the corresponding secondary antibodies conjugated with horseradish peroxidase at a 1:10 000 dilution in 5% skim milk (or 1:5 000 for phospho-ROCK-2 and p-AMPKα). Immobilon Forte Western HRP substrate (WBLUF0100, millipore, Etobicoke, ON, CA) was used to visualize immunoreactive bands and protein content was quantified using Computer-assisted densitometry ImageLab imaging software (Chemidoc, BioRad).

### 2.7. Animal and experimental design

C57Bl/6 male mice were purchased from Charles River (strain 027, Wilmington, MA, USA). At 8 weeks of age, the mice were rendered diabetic by intraperitoneal streptozotocin injection (50 mg/kg in 0.05 mol/L citrate buffer, pH 4.5) on 5 consecutive days after overnight fasting. Control mice were injected with citrate buffer. Blood glucose was measured with a glucometer (Contour, Bayer, Inc.) one week after the injections to confirm diabetes. All experiments were conducted in accordance with the Canadian Council of Animal Care and were approved by the Animal care and Use Committees of the Université de Sherbrooke, according to the NIH Guide for the Care and Use of Laboratory Animals.

### 2.8. Hindlimb ischemia model

After 2 months of diabetes, blood flow was measured in 16-week-old nondiabetic (NDM) and diabetic (DM) mice. Animals were anesthetized by inhalation of Isoflurane USP (1-chloro-2,2,2-trifluoroethyl difluoromethyl ether) at a concentration of 5% (initiation) and then maintained at 1%–2% during the whole surgical procedure (approximately 20 min). To mimic the ischemic condition in PAD, unilateral hindlimb ischemia was induced by ligation of the femoral artery as we previously described (24). Directly after the hindlimb surgery, osmotic pumps (model 1,004; Alzet Osmotic pumps, Cupertino, CA, USA) with Pyr-apelin-13 (in PBS) were implanted subcutaneously, according to the manufacturer's instructions, to allow 4-week infusions. Dose dependent experiments of Pyr-apelin-13 (0.36, 1 and 2 mg/kg/day) were performed in DM mice to select the proper concentration. The blood flow recovery following hindlimb ischemia was assessed in NDM, DM and DM + pyr-Ape-13.

### 2.9. Laser Doppler perfusion imaging

The hindlimb blood flow was measured using a laser Doppler perfusion imaging (PIMIII) system (Perimed Inc., Las Vegas, NV, USA). Consecutive perfusion measurements were obtained by scanning the region of interest (hindlimb and foot) of anesthetized animals. Measurements were performed pre-artery and post-artery ligation to ensure the success of the surgery, and on post-operative days 7, 14, 21 and 28. To account for variables that affect blood flow temporally, the results at any given time were expressed as a ratio against simultaneously obtained perfusion value of the ischemic (right) and nonischemic (left) hindlimb. Following laser Doppler perfusion imaging on day 28, mice were euthanized by exsanguination via the left ventricle under deep anesthesia (Isoflurane USP, inhalation at a concentration of 5%) and the ischemic adductor muscles were harvested.

### 2.10. Voluntary exercise wheel

Mice were housed individually for 5 consecutive days between day 23 and 27 to measure their activity using voluntary exercise wheel (Scurry mouse activity wheel, Model 80820FS, Lafayette Instrument Neuroscience, Lafayette, IN, USA). A magnetic sensor (Scurry mouse activity counter, Model 86,110) attached to the wheel recorded the number of revolutions and running distance using the Scurry activity monitoring software (Lafayette Instrument Neuroscience). Results are shown as cumulative distance of the 5 days.

### 2.11. Apelin peptide levels in the plasma

Mice were euthanized by exsanguination and blood was immediately centrifuged to isolate the plasma. Halt Protease Inhibitor Cocktail was added to the plasma to prevent apelin degradation and samples were frozen in liquid nitrogen. Peptides from the plasma were extracted on SEP-COLUMN containing 200 mg of C18 following the manufacturer's instructions. Briefly, the plasma was acidified using the Buffer A provided in the extraction kit and loaded into the pretreated C-18 SEP-COLUMNs. Columns were washed twice with Buffer A then the peptides were eluted with Buffer B. Eluants were evaporated to dryness in a centrifugal concentrator and then rehydrated in 125 μl of Assay Buffer provided in the EIA kit. Apelin-12 EIA kit was used according to the manufacturer's procedures to detect apelin-12, 13 and 36 isoforms, with a sensitivity level in the range of ng/ml, based on the principle of competitive EIA.

### 2.12. Histopathology

Ischemic adductor muscles from NDM, DM and DM + pyr-Ape-13 were harvested for pathological examination and sections were fixed in 4% paraformaldehyde (89,370–094, VWR, Radnor, PA, USA) for 18 h and then transferred to 70% ethanol. Fixed tissues were embedded in paraffin and 4 µm sections were stained with hematoxylin & eosin (H & E) or used for immunofluorescence assay. H & E-stained cross section images were captured with the Nikon eclipse Ti microscope, and 4 regions of 0.25 mm^2^ were randomly selected on the entire ischemic muscle. Within these areas, the diameter of 50 myofibers were randomly measured, using the Image J software, to obtain the mean myofiber diameter per mouse. All images were taken at the same time under identical settings.

### 2.13. Immunofluorescence

Cross-sections of ischemic adductor muscle were blocked at room temperature for an hour with 10% goat serum and then incubated overnight with primary antibodies (CD31 (1:50) and *α*-smooth muscle actin (α-SMA), 1:200), followed by 1 h incubation with the secondary antibody (1:400). Images of the entire ischemic muscle were taken with the Nikon eclipse Ti microscope. Vessels with only CD31-positive signal and a lumen diameter less than 10 µm (capillaries) were separately counted from vessels with CD31/α-SMA staining ranging from 10 to 30 µm (arterioles). Vascular density of capillaries and arterioles was normalized by the entire muscle fiber density of each mouse. All images were taken at the same time under identical settings and similarly handled in Adobe Photoshop and Image J software.

### 2.14. Quantitative PCR analysis

Quantitative PCR was performed to evaluate mRNA expression of genes in the ischemic adductor muscles and BAECs as we previously described (7). The expression of the housekeeping gene glyceraldehyde 3-phosphate dehydrogenase (GAPDH) was used to normalized data. The specific primer sequences are listed in Table 1.

**Table 1:** Sequences of primers.

Gene	Foward primer sequence	Reverse primer sequence
mAPJ	CAACCACAGCATGGGACAGAT	GGCTTGTCTCTCCCTGCTCTT
mApelin	CGCCCCTCACTTGGATGAT	AGGAGGATGGGCCAAAGG
meNOS	GTTTGTCTGCGGCGATGTC	GAATTCTCTGCACGGTTTGCA
mFLK-1	ATGTGAAGCCATCAACAAAGCGG	GGGCAGCAGGTTGCACAGTAATTT
mVEGF-A	GGAGTACCCCGACGAGATAGAGTA	AGCCTGCACAGCGCATC
mPDGFR-β	TCAGGGTTTTCCGCAATCAG	TCTGTTATTTTGCTGGACCCAAA
mPDGF-B	CACTTCCGGTTCATTTCTCTACCT	GAGCAGACTGAAGGGCACATG
mGAPDH	GCATGGCCTTCCGTGTTC	GATGTCATCATACTTGGCAGGTTT
bRhoA	GGGAGCTGGCCAAGATGAA	TTTGCCATATCTCTGCCTTCTTC
bNOS	CGGAACAGCACAAGAGTTACAAGAT	GTGTTGCTGGACTCCTTTCTCTTC
bGAPDH	TGGAAAGGCCATCACCATCT	GCATCACCCCACTTGATGTTG

Mus musculus (m), Bos taurus (b).

### 2.15. Statistical analyses

**Figure 1:**
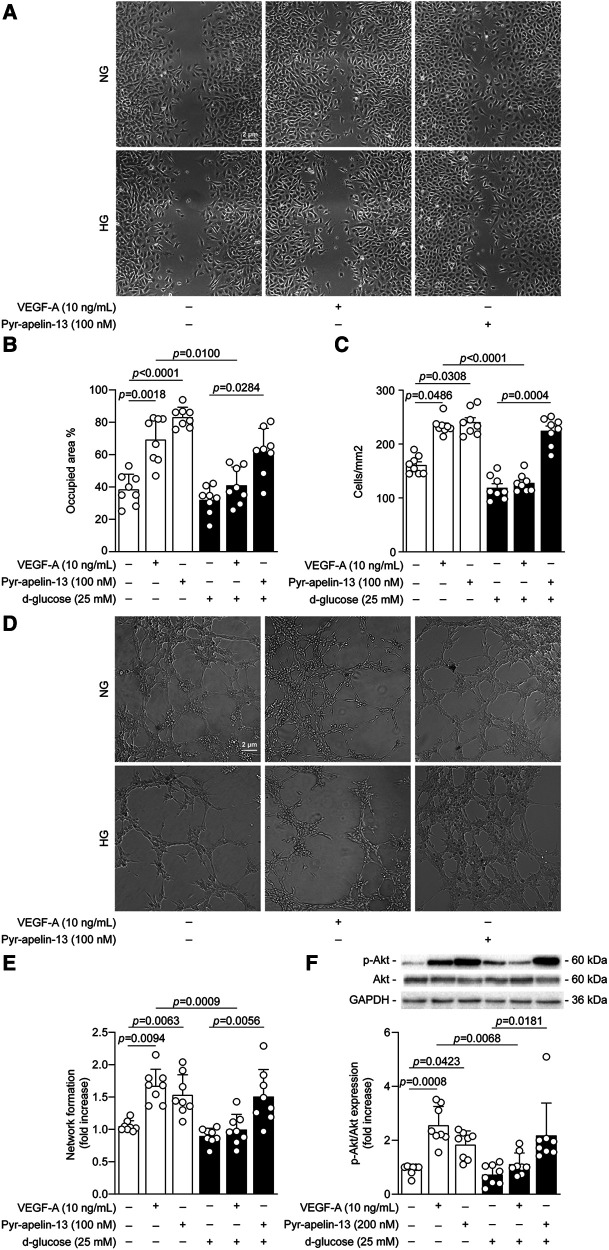
High glucose exposure did not affect Pyr-apelin-13 proangiogenic action in endothelial cells under hypoxia. BAECs were incubated with normal glucose (NG; 5.6 mmol/L; white bars) or high glucose (HG; 25 mmol/L; black bars) for up to 48 h and then stimulated with either VEGF-A or Pyr-apelin-13 for 16 h (**A–C**), 4 h (**D,E**), 5 min (VEGF) or 1 h (Pyr-apelin-13) (**F**) under hypoxia (1% O2) for the last 16 h of treatment in all experiments. (**A**) Representative images of the cell migration assay using the Ibidi's insert. (**B**) The percentage of the surface area occupied by the BAECs was quantified. (**C**) BAECs were fixed and stained with DAPI (4’,6-diamidino-2-phenylindole) and then cells were counted using the NIS-Elements software of Nikon eclipse Ti microscope. (**D**) Representative images of the lumen formation abilities of endothelial cells using Ibidi's μ-slide rack. (**E**) Tubule formation was quantified by measuring the total number of closed circles in the entire well, normalized on the NG condition. (**F**) Protein expression of Akt phosphorylation was detected by immunoblot analysis and the densitometry quantification was measured. Results are presented as the mean ± SD of 8 independent cell experiments.

**Figure 2:**
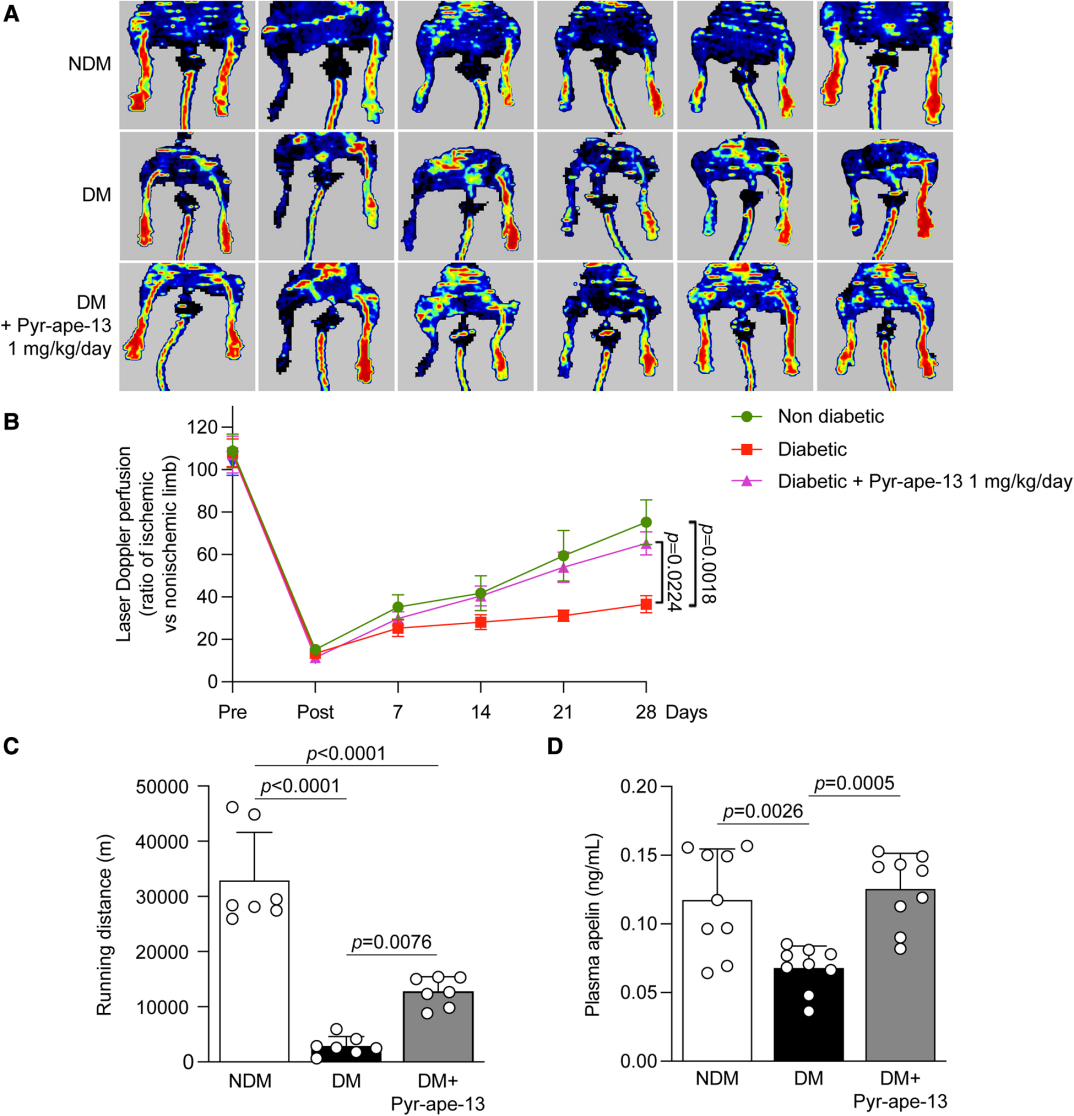
Pyr-apelin-13 delivery at 1 mg/kg/day improved blood flow reperfusion and enhanced running distance, vascular density and muscle structure in diabetic mice following critical limb ischemia. (**A**) Laser Doppler imaging and (**B**) blood flow reperfusion analysis of nondiabetic (NDM), diabetic (DM) and diabetic mice receiving Pyr-apein-13 (DM + Pyr-ape-13 1 mg/kg/day), pre, post, and 4 weeks following femoral artery ligation. (**C**) Cumulative running distance (m) over 5 days in the voluntary exercise wheel made by nondiabetic (NDM; white bars), diabetic (DM; black bars) and diabetic mice receiving Pyr-apelin-13 (DM + Pyr-ape-13; grey bars). (**D**) Apelin plasma levels in ng/ml. Results are presented as the mean ± SEM of 12 mice per group (**B**) and as the mean ± SD of 7 (**C**) and 9 (**D**).

**Figure 3:**
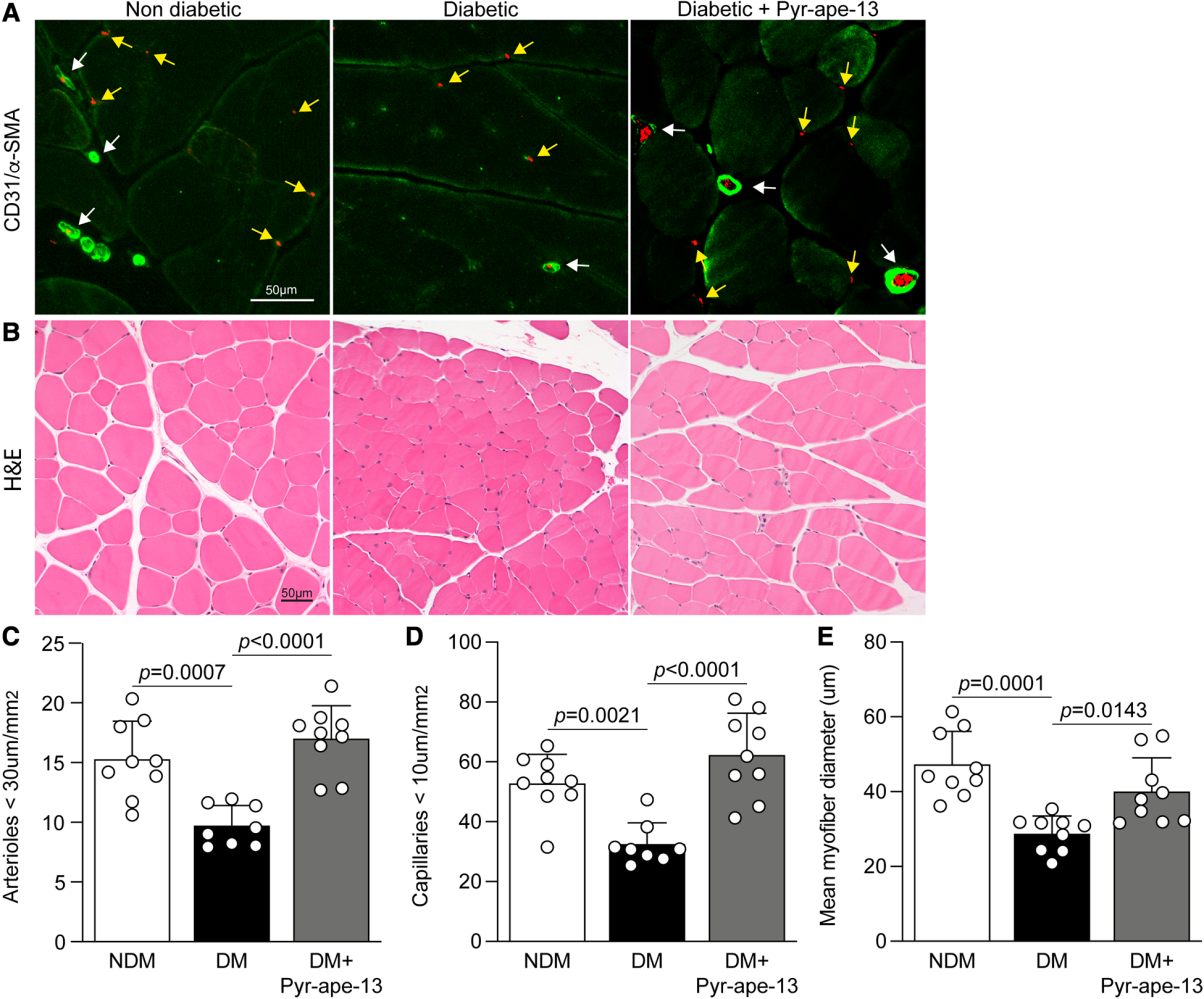
Pyr-apelin-13 administration in diabetic mice enhanced vascular density and improved muscle structure integrity following critical limb ischemia. (**A**) Immunofluorescence images of endothelial cells (CD31; red) and α-smooth muscle actin (α-SMA; green). The white arrows indicate vessels colocalized with α-SMA and CD31, and the yellow arrows indicate CD31-positive capillaries. (**B**) Morphological analysis of the ischemic muscle fiber stained with hematoxylin and eosin (H & E). (**C**) Quantification of the number of vessels smaller than 30 μm and (**D**) capillaries smaller than 10 μm in the ischemic muscle. (**E**) Quantification of the mean myofiber diameter in um of the ischemic muscle. Results are presented as the mean ± SD of 8-9 (**A,C,D**) and 9 mice per group (**B,E**).

**Figure 4:**
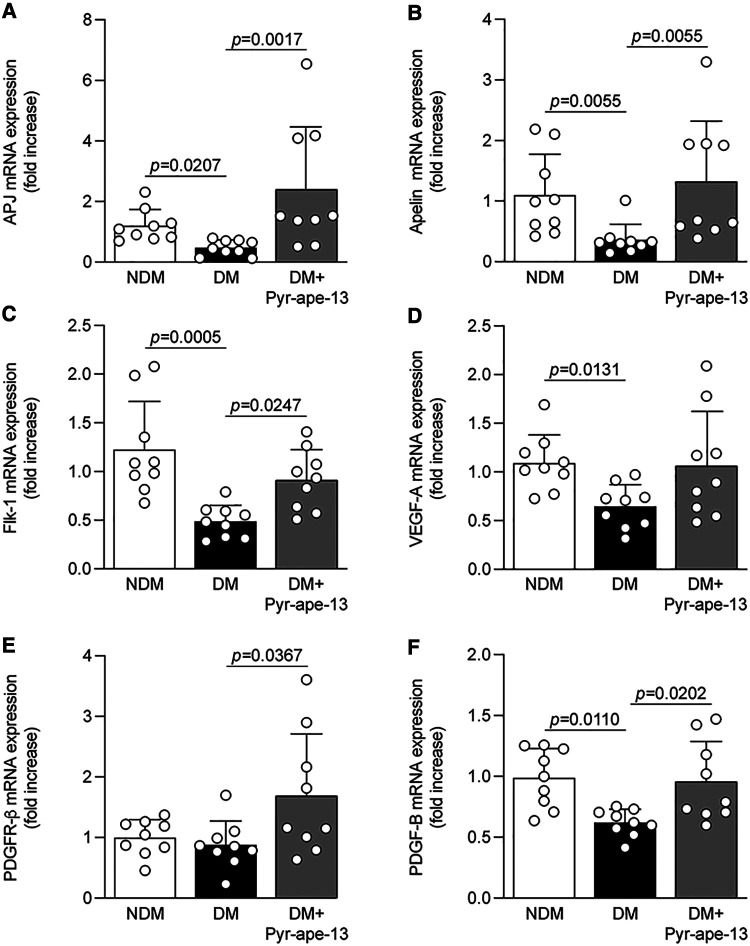
Pyr-apelin-13 increased the expression of growth factors in the ischemic muscle from diabetic mice following ischemia. Expression levels of (**A**) APJ, (**B**) Apelin, (**C**) Flk-1, (**D**) VEGF-A, (**E**) PDGFR-β and (**F**) PDGF-B mRNA in the ischemic muscle of nondiabetic (NDM; white bars), diabetic (DM; black bars) and diabetic mice receiving Pyr-apelin-13 (DM + Pyr-ape-13; grey bars). GAPDH gene was used for mRNA normalization. Results are presented as the mean ± SD of 9 mice per group.

**Figure 5:**
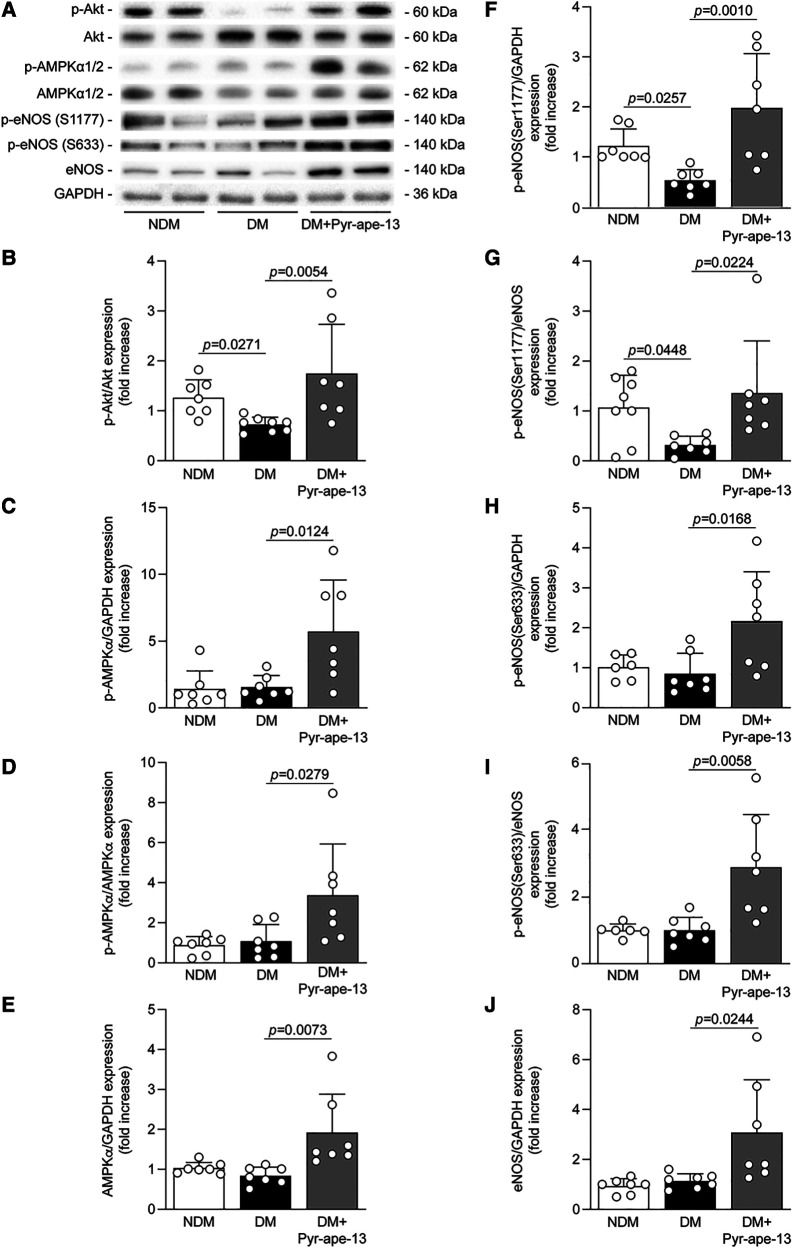
Pyr-apelin-13 delivery -induced angiogenesis following limb ischemia in diabetic mice is associated with the activation of Akt, AMPK and eNOS signaling pathways. (**A**) Protein expression of phospho-Akt, phospho-AMPKα1/2, AMPKα1/2, phospho-eNOS at Ser1177, phospho-eNOS at Ser633 and eNOS was detected by immunoblot analysis, and (**B–J**) densitometry quantification was measured, in the ischemic adductor muscle of nondiabetic (NDM; white bars), diabetic (DM; black bars) and diabetic mice receiving Pyr-apelin-13 (DM + Pyr-ape-13; grey bars). Results are presented as the mean ± SD of 7-8 (**B,G**), 7 (**C–F,J**), 6-7 (**H,I**) and 5–6 mice per group (**H**).

**Figure 6:**
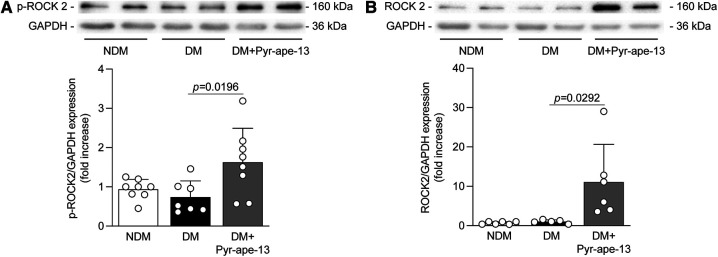
Pyr-apelin-13 delivery in diabetic mice promoted signaling pathway involved in cytoskeleton dynamics. Protein expression of (**A**) phospho-ROCK2 and (**B**) ROCK2 in the ischemic adductor muscle of nondiabetic (NDM; white bars), diabetic (DM; black bars) and diabetic mice receiving Pyr-apelin-13 (DM + Pyr-ape-13; grey bars) was detected by immunoblot analysis and the densitometry quantification was measured. Results are presented as the mean ± SD of 7-8 (**A**), and 5–6 mice per group (**B**).

**Figure 7:**
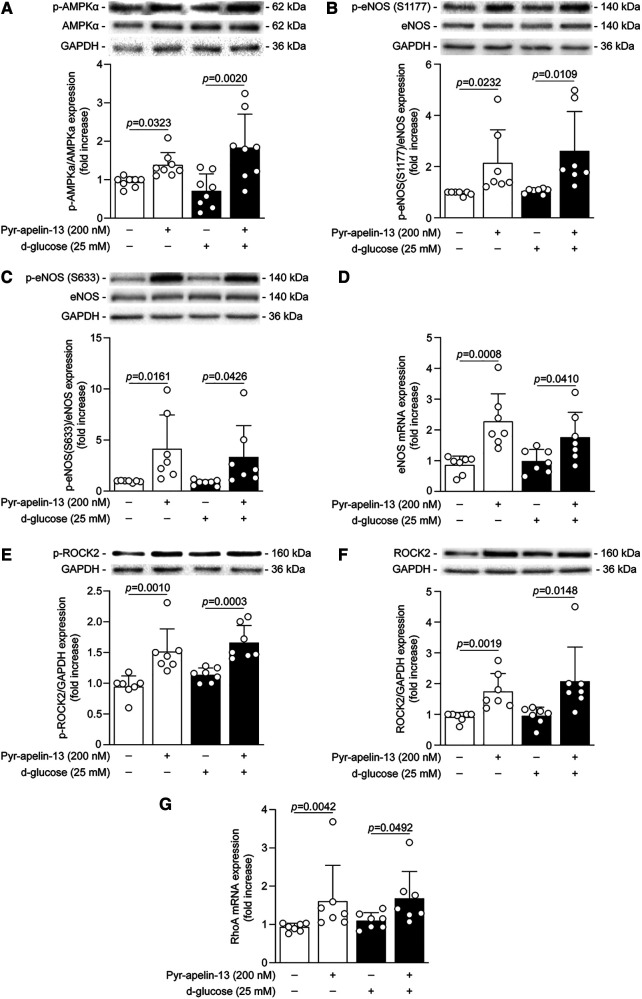
Pyr-apelin-13 enhanced the signaling pathways involved in cytoskeleton dynamics and proangiogenic actions in cultured endothelial cells exposed to high glucose conditions and hypoxia. BAECs were exposed to normal glucose (NG; 5.6 mmol/L; white bars) or high glucose (HG; 25 mmol/L; black bars) concentrations for 48 h and then stimulated with Pyr-apelin-13 for 1 h (**A–C,E,F**) or 24 h (**D,G**). BAECs were exposed to hypoxia (1% O2) for the last 16 h of treatment. Protein expression of (**A**) phospho-AMPKα1/2, (**B**) phospho-eNOS at Ser1177, (**C**) phospho-eNOS at Ser633, (**E**) phospho-ROCK-2 and (**F**) total protein expression of ROCK2 was detected by immunoblot analysis and the densitometry quantification was measured. mRNA levels of (**D**) eNOS and (**G**) RhoA. GAPDH gene was used for mRNA normalization. Results are presented as the mean ± SD of 8 (**A**) and 7 (**B-G**) independent cell experiments.

*In vivo* and *in vitro* data are shown as mean ± SD for each group except for blood flow measurements which are presented as mean ± SEM. Statistical analysis was performed by unpaired Kruskal–Wallis followed by Dunn's multiple comparisons test (Figures 1B,C,E,F, 4A–4D, 5G, 6A, 7G) or by unpaired One-Way ANOVA followed by Tukey's multiple comparisons test (Figures 2B–D, 3C–E, 4E,F, 5B–F,H–J, 6, 7A–F). Data in each group were checked for normal distribution using D'Agostino and Pearson normality test based on α = 0.05. All results were considered statistically significant at *P *< 0.05.

## 3. Results

### 3.1. Proangiogenic properties of Pyr-apelin-13 were not affected by high glucose exposure in endothelial cells

We have previously demonstrated that crucial factors involved in the angiogenic processes, such as VEGF, are affected in diabetes (7, 25). To determine and understand if apelin can improve endothelial function under a hyperglycemic milieu, we have evaluated its role on critical steps involved in blood vessel formation and compared its response to VEGF-A. Under NG conditions, hypoxia exposure promoted endothelial cell migration by 38% coverage of the surface area as compared to the surface area initially created by Ibidi's insert. As expected, under the same conditions, VEGF-A and Pyr-apelin-13 stimulation enhanced cell migration by 80% (*P *= 0.0018) and 116% (*P *< 0.0001), respectively (Figures 1A,B). As previously reported under HG and hypoxic conditions, VEGF-A-stimulated cell migration was inhibited by 92% (*P *= 0.0100), while Pyr-apelin-13 promoted endothelial cell migration by 93% (*P *= 0.0284; Figures 1A,B). Cell proliferation is also an important step in the angiogenic process. Our data demonstrated that the mitogenic effect of VEGF-A (1.4-fold; *P *= 0.0486) on endothelial cell proliferation under NG and hypoxic conditions were blunted in HG level exposure (Figure 1C). In contrast, Pyr-apelin-13-induced endothelial cell proliferation was conserved in HG conditions (1.9-fold; *P *= 0.004; Figure 1C). Then, we investigated the capacity of these growth factors to induce vascular lumen formation on Matrigel. We observed that VEGF and apelin treatment increased similarly endothelial cell lumen formation when exposed to NG condition and hypoxia by 1.6-fold (*P *= 0.0094) and 1.5-fold (*P *= 0.0063), respectively (Figures 1D,E). In line with the other angiogenic processes, the capacity of VEGF to induce lumen formation was abrogated in HG level exposure, which was not observed with Pyr-apelin-13. Treatment with Pyr-apelin-13 enhanced lumen formation in both NG (1.5-fold; *P *= 0.0063) and HG (1.7-fold; *P *= 0.0056) conditions (Figures 1D,E). Inhibition of VEGF-induced proangiogenic actions in diabetes has been well documented by us and others, and associated with impaired activation of VEGFR-2 and downstream effector Akt (7, 26). As we reiterated in our study, under NG and hypoxic conditions, VEGF stimulation led to a 2.8-fold increase (*P *= 0.0008) in Akt phosphorylation, whereas under HG exposure, Akt activation was reduced by 56% (*P *= 0.0068; Figure 1F). In contrast to VEGF-A, Pyr-apelin-13 stimulated Akt phosphorylation by 2-fold (*P *= 0.0423) and 3-fold (*P *= 0.0181) under hypoxia and NG or HG conditions, respectively (Figure 1F). Taking together, these results clearly demonstrate that HG conditions caused inhibition of VEGF actions on endothelial cell proangiogenic function, a phenomenon that was not observed under the same conditions following Pyr-apelin-13 stimulation.

### 3.2. Pyr-apelin-13 enhanced blood flow reperfusion following hindlimb ischemia in diabetic mice

Since *in vitro* experiences in endothelial cells exposed to HG levels indicated that treatment with apelin remained effective to induce proangiogenic actions, we investigated its potential beneficial effects in a diabetic hindlimb ischemia mouse model. Apelin has been shown to influence glucose and lipid metabolism as well as insulin resistance in animal models of type 2 diabetes (27). Here, treatment with apelin osmotic pumps did not influence systemic blood glucose nor affect weight in diabetic mice (Table 2). Because apelin's capacity to improve blood flow reperfusion following hindlimb ischemia in normoglycemic animals has already been reported (19, 22), but never in diabetic animals, we decided to only investigate its actions on diabetic mice. After 2 months of diabetes, femoral artery ligation was performed in nondiabetic and diabetic mice receiving or not systemic delivery of Pyr-apelin-13 with subcutaneous osmotic pumps. Blood flow reperfusion of the lower limb was measured once a week over a period of 4 weeks using laser Doppler imaging (Figure 2A and Supplementary Figure S1A). We selected 3 doses of Pyr-apelin-13 (0.36, 1 and 2 mg/kg/day) to determine which doses would induce the strongest blood flow recovery in diabetic mice. Dose dependent experiments indicated a highest blood flow reperfusion in diabetic mice receiving Pyr-apelin-13 at 1 mg/kg/day (Supplementary Figure S1B). Therefore, this concentration was used for further analysis. Following 28 days post-ligation, diabetic mice exhibited a 36% blood flow recovery compared to 75% in nondiabetic mice (*P *= 0.0018; Figure 2B). Interestingly, systemic administration of Pyr-apelin-13 in diabetic animals improved blood flow reperfusion to 65% (*P *= 0.0224; Figure 2B).

**Table 2:** Mean body weight and fasting glucose levels of mice.

	NDM	DM	DM + Pyr-apelin-13
Body weight (g)	29.9 ± 3.8	20.0 ± 2.8	22.4 ± 1.8
Blood glucose levels (mg/ml)	154.7 ± 24.7	513.6 ± 82.64	509.0 ± 84.89

Nondiabetic (NDM), diabetic (DM) and diabetic mice receiving Pyr-apelin-13 (DM + Pyr-apelin-13). Results are presented as the mean ± SD of 12 mice per group.

### 3.3. Pyr-apelin-13 improved motor function of the ischemic hindlimb in diabetic mice

A common symptom in patients with PAD is the development of fatigue or pain in the legs leading to reduced walking distance. Thus, we have measured the functional recovery by placing the mice in individual cages equipped with a voluntary exercise wheel, an environment without constraint and human intervention, during 5 consecutive days. Three weeks following limb ischemia, untreated diabetic mice exhibited a significant 90% reduction (*P *< 0.0001) in running distance as compared to nondiabetic mice (Figure 2C). Interestingly, diabetic mice receiving Pyr-apelin-13 improved by 4.4-fold (*P *= 0.0076) compared to untreated diabetic mice (Figure 2C). These results suggested an impact of diabetes on the functional recovery of the hindlimb following ischemia, that can be partially recovered by the administration of Pyr-apelin-13 in diabetic mice.

### 3.4. Diabetes reduced plasma apelin levels following ischemia

Apelin levels in the plasma were measured using Phoenix Pharmaceutical EIA kit, reported to cross-react with apelin-12, 13 and 36 isoforms. Several studies reported variations in apelin plasma concentrations in the context of diabetes (27, 28). We observed a 42% reduction (*P *= 0.0026) in apelin plasma levels in diabetic mice in response to limb ischemia compared to nondiabetic mice (Figure 2D). Implantation of Alzet osmotic pumps immediately after limb surgery ensured a continuous systemic delivery of apelin for a period of 28 days, which enhanced plasma apelin concentrations in diabetic mice by 1.8-fold (*P *< 0.0001) compared to untreated diabetic mice (Figure 2D).

### 3.5. Pyr-apelin-13 increased vascular density in the ischemic adductor muscle of diabetic mice

To support the blood flow reperfusion data, we evaluated the ability of Pyr-apelin-13 to promote collateral vessel formation following ischemia by measuring vascular density on cross-sections of the ischemic adductor and calf muscle of all groups of mice. Diabetes reduced the formation of arterioles (vessels <30 μm) and capillaries (CD31-positive vessels <10 μm) in response to tissue ischemia by 36% (*P *= 0.0007; Figures 3A,C) and 38% (*P *= 0.0021; Figures 3A,D), respectively, compared to normoglycemic littermate controls. However, delivery of Pyr-apelin-13 in diabetic mice significantly enhanced the amount of arterioles, from 9.71 to 17.00 vessels/mm^2^ (1.8-fold, *P *< 0.0001; Figures 3A,C), as well as the formation of capillaries, raised from 32.55 to 62.26 (1.9-fold, *P *< 0.0001; Figures 3A,D), suggesting that Pyr-apelin-13 treatment can promote angiogenesis/arteriogenesis in response to ischemia despite being exposed to diabetes. Hindlimb ischemia causes structural disorganization and degeneration of the muscle fiber (29), and muscle fiber atrophy has been observed in biopsy from patients with PAD (30). We measured myofiber diameter on H & E staining sections of the ischemic muscle as an indicator of muscle fiber atrophy. As a result of persistent ischemia in diabetic mice hindlimb, we observed a 39% reduction (*P *= 0.0001; Figures 3B,E) in mean myofiber size compared to nondiabetic mice. Interestingly, apelin delivery enhanced mean myofiber diameter by 1.4-fold (*P *= 0.0143; Figures 3B,E), which was associated with improvement of blood flow reperfusion in diabetic mice.

### 3.6. Treatment with Pyr-apelin-13 enhanced growth factor expression in diabetic ischemic hindlimb

Apelin and its receptor are known to be upregulated in the muscle and the artery by hypoxia in nondiabetic animal models (21, 22). Gene expression of the apelinergic system is influenced by diabetes in different tissues (27, 31). Following 28 days of femoral artery ligation, we observed that diabetes reduced mRNA expression of both APJ and apelin in the ischemic muscle by 60% (*P *= 0.0207; Figure 4A) and 67% (*P *= 0.0055; Figure 4B), respectively. In contrast, Pyr-apelin-13 administration increased APJ mRNA expression by 5-fold (*P *= 0.0017; Figure 4A) and its own expression by 3.6-fold (*P *= 0.0055; Figure 4B) in diabetic mice. As we previously reported, many other growth factors are downregulated by diabetes in the ischemic muscle, including VEGF-A, Flk-1, PDGF-B and PDGFR-β (7, 8, 24). Increased capillary density in the ischemic hindlimb of diabetic mice receiving Pyr-apelin-13 led us to investigate whether apelin influenced the expression pattern of well-known proangiogenic growth factors. In our study, Flk-1 (*P *= 0.0005; Figure 4C), VEGF-A (*P *= 0.0131; Figure 4D) and PDGF-B (*P *= 0.0110; Figure 4F) mRNA expression in the ischemic muscle were significantly decreased by diabetes as compared to nondiabetic controls. Our results indicated that the administration of Pyr-apelin-13 significantly increased the gene expression of Flk-1 (*P *= 0.0247; Figure 4C), VEGF-A (*P *= 0.0746; Figure 4D), PDGFR-β (*P *= 0.0367; Figure 4E) and PDGF-B (*P *= 0.0202; Figure 4F) in diabetic mice.

### 3.7. Pyr-apelin-13-induced revascularization *in vivo* is associated with proangiogenic and cytoskeleton organization signaling pathways

The signaling pathways by which apelin may promote angiogenesis under hyperglycemia were investigated in the ischemic adductor muscle. The binding of apelin to its receptor APJ leads to the recruitment of the small Gα_i_ protein which subsequently activates the Akt signaling pathway, promoting cell survival and angiogenesis. As expected, diabetes markedly reduced Akt phosphorylation in the ischemic muscle by 42% (*P *= 0.0271) compared to nondiabetic mice (Figures 5A,B). In contrast, diabetic mice who received Pyr-apelin-13 exhibited a 2.4-fold (*P *= 0.0054) increase Akt phosphorylation (Figures 5A,B). APJ activation may also induce the phosphorylation and activation of AMPK via the Gα_i_ and Gα_q_ subunits (32). AMPK is mainly known as a fuel-sensing enzyme and for its metabolic function, but it also promotes endothelial cell proliferation and migration (33). Our results showed that apelin delivery raised AMPKα1/2 phosphorylation by 3.1-fold (*P *= 0.0279; reported to total AMPK) to 3.7-fold (*P *= 0.0124; reported to GAPDH) (Figures 5A,C,D) and total protein expression by 2.2-fold (*P *= 0.0369; Figures 5A,E) in the ischemic muscle of diabetic mice compared to untreated diabetic mice. Then, we assessed eNOS as a common downstream target of Akt and AMPK. Once phosphorylated, eNOS produces NO, a major regulator of endothelial cell growth and angiogenesis (34). It has been shown that Ser 633 and Ser1177 of eNOS can be phosphorylated by AMPK while Akt only phosphorylates eNOS at Ser1177 (35, 36). As expected, diabetes reduced Ser1177 phosphorylation of eNOS by 57% (*P *= 0.0257; reported to GAPDH) and 70% (*P *= 0.0448; reported to total eNOS) (Figures 5A,F,G) compared to normoglycemic animals but had little impact on eNOS Ser633 activation. Interestingly, the administration of Pyr-apelin-13 in diabetic mice increased both Ser1177 phosphorylation of eNOS by 3.7-fold (*P *= 0.0010; reported to GAPDH) or 4.3-fold (*P *= 0.0224; reported to total eNOS) (Figures 5A,F,G) and Ser633 by 2.5-fold (*P *= 0.0168; reported to GAPDH) or 2.1-fold (*P* = 0.0058; reported to total eNOS) (Figures 5A,H,I). Furthermore, apelin delivery in diabetic mice elevated total eNOS protein expression in the ischemic muscle by 2.7-fold (*P *= 0.0244) compared to untreated diabetic mice (Figures 5A,J). We also explored the RhoA/ROCK signaling pathway, which is known to be recruited by the small Gα_12/13_ protein following GPCRs activation (37), and can mediate cellular morphological processes such as cell contractility, actin cytoskeleton organization, cell migration and proliferation (38). Although no difference in ROCK-2 phosphorylation and protein expression was observed in the ischemic muscle of diabetic mice compared to nondiabetic mice, apelin administration raised ROCK2 phosphorylation by 2.2-fold (*P *= 0.0196; Figure 6A) and ROCK-2 total protein expression by 10.7-fold (*P *= 0.0292; Figure 6B) compared to untreated diabetic mice. We did not observe any difference in phospho-ROCK2 protein expression when reported on ROCK2 expression (Supplementary Figure S2A), due to significant increased expression of ROCK-2 in diabetic mice receiving Pyr-apelin-13.

### 3.8. Pyr-apelin-13 enhanced cultured endothelial cells proangiogenic functions through the Akt/AMPK/eNOS and RhoA/ROCK2 pathways

To confirm that the signaling pathways enhanced by the treatment with Pyr-apelin-13 in ischemic muscle occurred in endothelial cells, we investigated their activation in cultured endothelial cells exposed to hypoxia and NG or HG conditions. Either under NG and HG exposure, 1 h stimulation with Pyr-apelin-13 similarly raised AMPKα1/2 (Figure 7A) and eNOS phosphorylation at Ser1177 (Figure 7B) and Ser633 (Figure 7C). In addition, eNOS mRNA expression in endothelial cells was increased by 2.6-fold (*P *= 0.0008) and 1.8-fold (*P *= 0.0410) following 24 h stimulation of apelin under NG and HG conditions, respectively (Figure 7D). As for the *in vivo* results, we observed a very similar pattern for RhoA/ROCK2 signaling pathway, apelin treatment increased ROCK-2 phosphorylation (Figure 7E) and total protein expression (Figure 7F) as well as RhoA mRNA expression (Figure 7G) in both NG and HG conditions. However, no significant difference in ROCK-2 phosphorylation following apelin stimulation when reported on ROCK-2 protein expression was noted (Supplementary Figure S2B). These data demonstrate that apelin stimulation, through APJ receptor, promotes signaling pathways endothelial cells that are not affected by hyperglycemia.

## 4. Discussion

Patients with diabetes and PAD are 5 to 15 times more likely to undergo major amputation due to vascular abnormalities (39). Indeed, diabetes blunts the angiogenic response, leading to impaired wound healing and collateral vessel formation (40). Endogenous apelin has been shown to participate in the collateral vessel formation processes during ischemia (21). Previous studies reported that apelin treatment or apelin gene therapy improved limb reperfusion after femoral artery ligation but only in nondiabetic animal models (22, 23). Very few studies have characterized the impact of the apelinergic system on the angiogenic response under diabetic conditions. Our study demonstrated for the first time that apelin treatment enhanced endothelial cell function under hypoxia and high glucose exposure as well as improved blood flow reperfusion and functional recovery of the hindlimb following ischemia in a diabetic condition.

The vascular endothelial growth factor (VEGF) is one of the most extensively studied growth factors in relation to physiological angiogenesis. Although preclinical studies using VEGF therapies in animal models of hindlimb ischemia were promising (41), all phase II clinical trials failed to significantly reduce the amputation rate in patients suffering of PAD (42). A possible reason for these clinical disappointments may be caused by a reduction in VEGF receptors and downstream signaling (Akt, eNOS) activation in response to ligand binding created by high glucose exposure (7). As we demonstrated previously, diabetes increased the activity of the SH2 domain-containing phosphatase 1 (SHP-1) and its binding with the receptors tyrosine kinase (RTK) VEGFR-2 and PDGFR-β, reducing receptor activity and downstream signaling pathways associated with proangiogenic actions (7, 24). The present study corroborated these findings that hyperglycemia prevented the activation and phosphorylation of VEGFR-2 downstream effector Akt, the proliferation, migration and lumen formation in response to VEGF stimulation in endothelial cells. In our study, we have recreated the ischemic state found in PAD and observed that Pyr-apelin-13 stimulation in endothelial cells exposed to the combination of hypoxia and NG or HG concentrations enhanced Akt and eNOS phosphorylation, promoted cell proliferation, migration and lumen formation. Moreover, our results indicated that Pyr-apelin-13 delivery in diabetic mice markedly increased Akt and eNOS phosphorylation, as well as eNOS protein expression in ischemic muscle. Interestingly in a model of infarcted myocardium in diabetic rats, *Azizi and al*. also reported that apelin treatment increased eNOS expression (43). VEGFR2 (Flk-1) and PDGFR-β are not direct targets of APJ/apelin signaling pathways, but interestingly, we identified that apelin administration increased VEGF-A, VEGFR-2, PDGF-B and PDGFR-β mRNA expression in diabetic ischemic muscle. Previous study showed that apelin treatment can restore VEGFR2 expression and enhance cardiac endothelial cell proliferation and migration exposed to high glucose levels (44). However, these experiments were performed under normoxia, which may not reflect the ischemic condition in PAD. Taken together, our data suggest that Pyr-apelin-13 delivery circumvents the resistance mechanism induced by hyperglycemia, providing an effective angiogenic response under hypoxic conditions.

The apelinergic system's implication in angiogenesis has been demonstrated by the presence of apelin/APJ in tip cells and stalk cells, which are responsible for guiding the new vessel formation toward the gradient of proangiogenic factors and for the elongation of the sprouting vessel (45). Furthermore, other studies reported apelin/APJ upregulated expression under hypoxic and normoglycemic conditions in cultured vascular cells and peripheral mononuclear cells, as well as in mouse lungs following ischemia (46). The presence of hypoxia response element (HRE) sequence was found on apelin and APJ genes, making them targets of hypoxia-inducible factor 1 (HIF-1). Few studies investigated the basal level of expression of the apelinergic system in nonischemic tissues from diabetic animals and reported a decreased expression of apelin and/or APJ in the heart and skeletal muscle (27, 31). However, the relationship between diabetes and apelin plasma levels remains to be clarified. Some studies reported increased plasma apelin concentrations in obese and type 2 diabetic patients and mice (31, 47, 48), while others have shown decreased levels in these conditions (27, 49). In our study, we used a type 1 diabetes mouse model, and our results demonstrated that despite the presence of tissue ischemia, diabetes prevented the basal expression of APJ/apelin in the adductor muscle and lowered plasma apelin concentrations compared to nondiabetic mice. Interestingly, we showed that administration of Pyr-apelin-13 in diabetic animals enhanced APJ and apelin expression, improving endothelial function and angiogenesis. Collectively, these data suggest that the repressive effect of diabetes on the apelinergic system expression can be overcome by apelin administration, resulting in a proper recovery from tissue ischemia.

AMPK is a metabolite-sensing protein kinase mostly known for its metabolic effects, but several studies emphasized its role during angiogenesis. Indeed, *Nagata and al.* reported that AMPK activation is essential for angiogenesis under hypoxic conditions, but dispensable to the angiogenic response under normoxic conditions (50). As Akt, AMPK mediates the activity of eNOS by phosphorylation at Ser1177, which characterized its activation state, to induce NO release, promoting vasodilatation and proangiogenic actions (34). Interestingly, AMPK but not Akt is able to phosphorylate eNOS in Ser633, a phosphorylation site reported to be an effective indicator of eNOS-induced NO bioavailability, resulting in enhanced endothelial cell migration and tubule formation (35). Apelin has been reported to induce AMPK, Akt and eNOS phosphorylation under normal glucose concentrations (51). Here, we demonstrated that these effects were preserved in hypoxic endothelial cells and ischemic muscle following apelin stimulation despite being exposed to HG levels and diabetes. Taken together, our results demonstrated that apelin treatment, under hypoxic and HG or diabetic conditions, induced eNOS activation and proangiogenic actions through two different phosphorylation sites involving Akt and AMPK activation.

Endothelial cell mobility involves the sensing of proangiogenic signals by filopodia, the formation, protrusion and extension of cytoskeleton projections, the assembly/disassembly of focal adhesions, the contraction of the cell body enabling forward movement and rear release. All these processes are mediated by the Rho small GTPase family and its protein kinase effector ROCK (52), a classical pathway downstream of the small Gα_12/13_ protein recruitment following GPCRs activation (37). ROCK protein kinases are necessary for normal angiogenesis during embryonic development since ROCK1 or ROCK2 knockout mice died *in utero* due to decreased vasculature development (53). Furthermore, inhibition of RhoA/ROCK signaling pathway suppresses VEGF-induced endothelial cell migration and tube formation, and angiogenesis *in vivo* (54). Compared to ROCK1, ROCK2 is preferentially expressed in vascular cells (55), and gene knockdown of ROCK2, but not ROCK1, reduced endothelial cell tube formation *in vitro* and vascular density in mouse lungs (54). However, no study demonstrated concrete evidence of the activation of this signaling pathway following apelin stimulation. To our knowledge, we demonstrated for the first time that apelin stimulation, through APJ receptor, led to the activation of the RhoA/ROCK signaling pathway in the ischemic muscle from diabetic mice and in endothelial cells exposed to HG levels. These results highlight the role of the apelinergic system on the actin cytoskeleton remodeling in endothelial cells, which potentially facilitate angiogenesis and arteriogenesis following ischemia.

Pain in the legs, also called claudication, is a classic symptom of PAD. It is caused by the narrowing of arteries which reduces the blood flow to the leg, causing pain or cramp when walking. As the disease progress, claudication considerably decreases patient walking distance, up to prevent them from walking due to the severity of the pain. Our results clearly demonstrated the association between poor blood flow and reduced walking distance since diabetic mice displayed a 39% lower blood flow recovery and a 91% decreased running distance compared to nondiabetic controls. The increased blood flow reperfusion we observed following apelin treatment was supported by an increase in small vessel density in the ischemic muscle compared to untreated diabetic mice. Here, we demonstrated for the first time, that sustained apelin delivery improved blood flow reperfusion in diabetic mice and resulted in enhanced walking distance, which is an important clinical endpoint. Despite the improved functional recovery of the limb with apelin, an important gap remains compared to nondiabetic mice. One hypothesis could be nerve degeneration caused by diabetes, leading to typical symptoms of peripheral neuropathy such as muscle weakness, extreme sensitivity, and numbness. Interestingly, a recent study reported apelin's neuroprotective and regenerative effects in a nondiabetic focal cerebral ischemia mouse model (56). However, further investigations will be required to determine the potential effects of apelin in diabetic neuropathy.

Apelin proangiogenic actions in normoglycemic conditions are well documented. Our study demonstrated that, unlike VEGFR signaling pathways, the apelin/APJ pathways are not affected by hyperglycemia, making the apelinergic system a potential target for angiogenic therapy. In addition, we demonstrated the therapeutic effect of Pyr-apelin-13 in diabetic PAD, since its prolonged administration resulted in improved blood flow reperfusion, vascular density and motor function in diabetic mice following hindlimb ischemia. However, since VEGF therapy showed promising results in preclinical studies but was unsuccessful in clinical trials, it remains to be proven if apelin treatment can be an effective therapy for patients with diabetes and PAD. Furthermore, apelin's short plasma half-life may represent a challenge for its use as a pharmacological treatment. Thus, research must continue to develop more stable apelinergic agonists to improve the long-term angiogenic response in diabetes (57).

## Data Availability

The original contributions presented in the study are included in the article/Supplementary Material, further inquiries can be directed to the corresponding author.
