# Supplementary material for: Apelin prevents diabetes-induced poor collateral vessel formation and blood flow reperfusion in ischemic limb
Source: Front Cardiovasc Med. 2023 Aug 11;10:1191891. doi: 10.3389/fcvm.2023.1191891 (PMC10450936; doi:10.3389/fcvm.2023.1191891)
Supplement: Supplementary file 1 [file Datasheet1.pdf]

Supplementary Material

Apelin prevents diabetes-induced poor collateral vessel formation and blood flow reperfusion in ischemic limb

Stéphanie Robillard, Kien Trân, Marie-Sophie Lachance, Tristan Brazeau, Elizabeth Boisvert, Farah Lizotte, Mannix Auger-Messier, Pierre-Luc Boudreault, Éric Marsault, and Pedro Geraldes*.

*** Correspondence:** Pedro Geraldes: Pedro.Geraldes@USherbrooke.ca

1 Supplementary Data

2 Supplementary Figures and Tables

2.1 Supplementary Figures

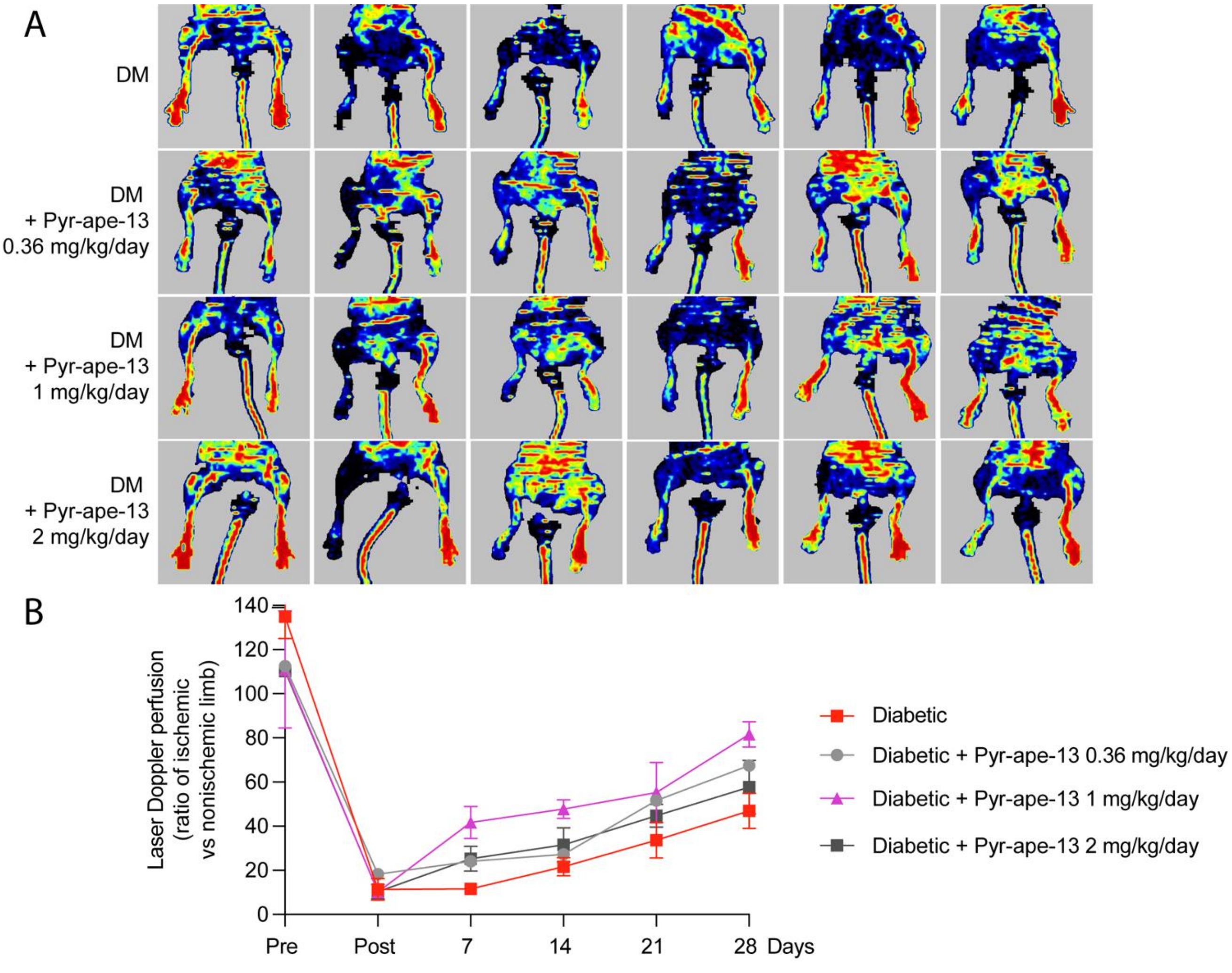

Supplementary Figure 1. (A) Laser Doppler imaging and (B) blood flow reperfusion analysis of diabetic (DM) and diabetic mice receiving different doses of Pyr-apein-13 (DM+Pyr-ape-13 0.36, 1 or 2 mg/kg/day), pre, post, and 4 weeks following femoral artery ligation. Results are presented as the mean ± SEM of 1-2 mice per group.

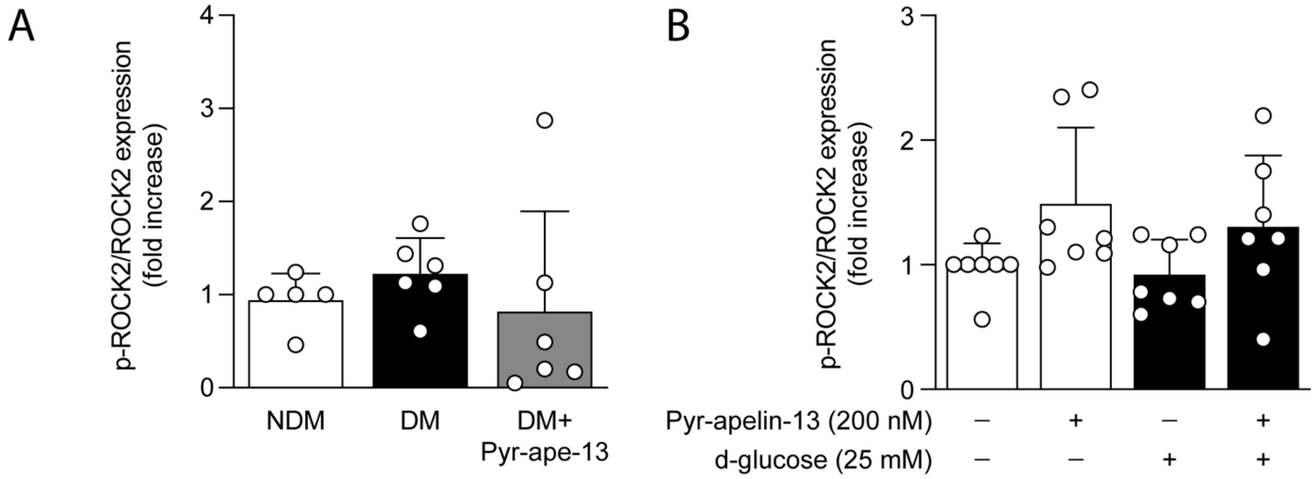

Supplementary Figure 2. Densitometry quantification of immunoblot analysis representing phospho-ROCK-2 protein expression reported on ROCK-2 expression *in vivo* and *in vitro*. (**A**) Phospho-ROCK-2 protein expression in the ischemic adductor muscle of nondiabetic (NDM; white bars), diabetic (DM; black bars) and diabetic mice receiving Pyr-apelin-13 (DM+Pyr-ape-13; grey bars). (B) Phospho-ROCK-2 protein expression in cultured BAECs exposed to normal glucose (NG; 5.6 mmol/L; white bars) or high glucose (HG; 25 mmol/L; black bars) concentrations for 48h, to hypoxia (1% O_2) for the last16h of treatment and then stimulated with Pyr-apelin-13 for 1h. Results are presented as the mean ± SD of 5-6 mice per group (**A**) and 7 (**B**) independent cell experiments.
